# Differential Responses to Aging Among the Transcriptome and Proteome of Mesenchymal Progenitor Populations

**DOI:** 10.1093/gerona/glae147

**Published:** 2024-06-05

**Authors:** Jack Feehan, Nicholas Tripodi, Dmitry Kondrikov, Tissa Wijeratne, Jeffrey Gimble, William Hill, Vasso Apostolopoulos, Gustavo Duque

**Affiliations:** Department of Medicine—Western Health, University of Melbourne, Melbourne, Victoria, Australia; Institute for Health and Sport, Victoria University, Melbourne, Victoria, Australia; Institute for Health and Sport, Victoria University, Melbourne, Victoria, Australia; Australian Institute for Musculoskeletal Science (AIMSS), Western Health, Victoria University and University of Melbourne, Melbourne, Victoria, Australia; Department of Pathology and Laboratory Medicine, Medical University of South Carolina, Charleston, South Carolina, USA; Department of Medicine—Western Health, University of Melbourne, Melbourne, Victoria, Australia; Australian Institute for Musculoskeletal Science (AIMSS), Western Health, Victoria University and University of Melbourne, Melbourne, Victoria, Australia; Center for Stem Cell Research and Regenerative Medicine, Tulane University School of Medicine, New Orleans, Louisiana, USA; Department of Veterans Affairs, Ralph H Johnson VA Medical Center, Charleston, South Carolina, USA; Center for Healthy Aging, Medical University of South Carolina, Charleston, South Carolina, USA; Institute for Health and Sport, Victoria University, Melbourne, Victoria, Australia; Australian Institute for Musculoskeletal Science (AIMSS), Western Health, Victoria University and University of Melbourne, Melbourne, Victoria, Australia; Bone, Muscle & Geroscience Research Group, Research Institute of the McGill University Health Centre, Montreal, Quebec, Canada; Department of Medicine, McGill University, Montreal, Quebec, Canada

**Keywords:** Adipose-derived stem cells, Circulating osteoprogenitors, Geroscience, Mesenchymal stem cells, Stem cells

## Abstract

The biological aging of stem cells (exhaustion) is proposed to contribute to the development of a variety of age-related conditions. Despite this, little is understood about the specific mechanisms which drive this process. In this study, we assess the transcriptomic and proteomic changes in 3 different populations of mesenchymal progenitor cells from older (50–70 years) and younger (20–40 years) individuals to uncover potential mechanisms driving stem cell exhaustion in mesenchymal tissues. To do this, we harvested primary bone marrow mesenchymal stem and progenitor cells (MPCs), circulating osteoprogenitors (COP), and adipose-derived stem cells (ADSCs) from younger and older donors, with an equal number of samples from men and women. These samples underwent RNA sequencing and label-free proteomic analysis, comparing the younger samples to the older ones. There was a distinct transcriptomic phenotype in the analysis of pooled older stem cells, suggestive of suppressed proliferation and differentiation; however, these changes were not reflected in the proteome of the cells. Analyzed independently, older MPCs had a distinct phenotype in both the transcriptome and proteome consistent with altered differentiation and proliferation with a proinflammatory immune shift in older adults. COP cells showed a transcriptomic shift to proinflammatory signaling but no consistent proteomic phenotype. Similarly, ADSCs displayed transcriptomic shifts in physiologies associated with cell migration, adherence, and immune activation but no proteomic change with age. These results show that there are underlying transcriptomic changes with stem cell aging that may contribute to a decline in tissue regeneration. However, the proteome of the cells was inconsistently regulated.

All mesenchymal tissues, such as bone, fat, muscle, and cartilage, undergo a constant process of repair and renewal over the lifespan (1). In most instances, this regeneration is driven by a balance between anabolic development and catabolic breakdown of the tissue. As an individual age, however, these processes are commonly dysregulated, with the balance between anabolism and catabolism being lost. This is seen in many tissues and leads to some of the most prevalent aging-related diseases, including musculoskeletal conditions such as osteoporosis and sarcopenia (2,3).

The modern field of geroscience seeks to develop a unifying paradigm of physiological changes that occur in aging, which drive the onset of these diseases (4). Geroscience has described key physiologies, such as inflammaging, altered proteostasis, genetic damage, and epigenetic changes as collectively underpinning these diseases (5,6). In addition, another key pillar in musculoskeletal and other degenerative conditions is stem cell exhaustion (7), which is a process by which stem cells lose or alter their multipotency and are less able to maintain tissue quality with increasing age.

Mesenchymal tissue regeneration is mediated by a range of progenitor cells, of which the best understood is the mesenchymal stem and progenitor cells population (MPC), which resides in the perivascular niche in the bone marrow (1). Once known as mesenchymal stem cells (MSCs), and thought to be a distinct entity, they are now more accurately characterized as a heterogenous population, although the roles of their subpopulations are poorly understood (8). Additionally, in recent years, cells with a capacity for mesenchymal differentiation have been identified in various tissues, including the circulation and adipose tissue. The adipose-derived adult stem cell (ADASC) is a plastic adherent, mesenchymal lineage-restricted stem cell population derived from brown and white adipose tissue (9). These cells are similar in many regards to the bone marrow MPCs, both phenotypically and functionally, but are also involved in regulating energy metabolism due to their localization to adipose tissue. The circulating osteoprogenitor (COP) cell is a newly discovered mesenchymal progenitor in the peripheral circulation of adults (8,9). Once thought to simply be a bone marrow MPC stimulated to circulate, following the discovery of the bone marrow as their origin through parabiosis experiments (10), they have since been shown to be phenotypically distinct bearing markers of the hematopoietic lineage. Although now known to be a specific population of cells, they have been suggested to differentiate along mesodermal lineages and contribute to several age-related diseases, including osteoporosis (11), fracture healing (12), and heterotopic ossification (13). Although there remain significant unknowns about these progenitor populations, they have generated substantial interest in the field of regenerative medicine, with several of them under investigation for therapeutic use in several disease settings, including osteoporosis, sarcopenia, and fracture. However, given these conditions most commonly onset in older age, there is a great need to understand how they are affected by aging.

Mesenchymal stem and progenitor cells, ADASCs, and COP cells have been associated with both physiological and pathological tissue development. However, there are still significant unknowns regarding their relationships and specific roles. It has been suggested that all 3 changes with age and that these changes may contribute to the development of musculoskeletal diseases. For example, MPCs isolated from older individuals have a decreased capacity to form bone, an increased capacity for adipogenesis, and altered miRNA and epigenetic regulation (14,15). Interestingly, it has been repeatedly shown that aging MPCs have altered immunoregulatory function, with transcriptomic analyses showing decreased expression of GATA2 and PD-L1 (16). COP cell numbers are associated with age, bone density, and vitamin D status in older adults (11,17), and ADASCs become prooxidative and proinflammatory (18). Although these physiological outcomes have been shown, there is little understanding of the specific mechanisms driving these changes. It is also unclear whether there is a common physiological driver of these changes across all adult stem cell populations, or whether each group of progenitors is affected differently. Therefore, we sought to contrast these 3 groups of progenitors with samples taken from younger and older adults to identify and compare the genetic and proteomic alterations in stem and progenitor cell aging.

## Method

### Biosafety and Ethics

This study was undertaken in laboratories at the Medical University of South Carolina, Charleston, SC, USA, and the University of Melbourne, Victoria, Australia. All experiments were undertaken with aseptic procedures, under appropriate biosafety conditions. All human samples were collected following Human Research Ethics Approval at the site of collection. The COP cell samples were acquired from the Australian Red Cross Blood Service (ARCBS), following a waiver of ethics requirements from the Western Health Human Research Ethics Committee.

### Primary Cell Cultures

The experiments in this study were undertaken on primary human musculoskeletal progenitor cells taken from donors from specific age and sex groups. For each primary cell type (COP, ADSC, and MPCs), there were 16 total samples—4 taken from younger (18–40) men, 4 from younger women, 4 from older (50–80) men, and 4 from older women, for a total of 48 samples which underwent analysis. Samples from each of the 48 donors underwent both transcriptomics and proteomics, to allow more accurate alignment of the data sets.

### COP Cells

Circulating osteoprogenitor cells were acquired as documented in a previous validation study (19). Briefly, buffy coats were obtained from therapeutic blood donations from the ARCBS, and white cells were purified through Ficoll density gradient separation (GE Healthcare Companies, IL, USA, GE17-1440-02). Once purified buffy coats were collected, fluorescence-activated cell sorting was used to isolate the COP cells. The isolated leukocytes were incubated with FcR blocking reagent (Miltenyi Biotec, Germany, Cat. No: 130-059-901) for 10 minutes before being labeled with the conjugated fluorescent antibodies (CD45- Fluorescein isothiocyanate [BD Biosciences, NJ, USA, Cat. No: 555482], ALP-brilliant violet 421 Allophycocyanin [BD Biosciences, NJ, USA, Cat. No: 752998], and CD34-Allophycocyanin [BD Biosciences, Cat. No: 340441]) at a concentration of 1:100 v/v in the dark at 4 °C for 30 minutes. Cells were then costained with a viability dye (7-AAD [BD-Biosciences, Cat. No: 559925]) and processed on a 4 laser (405 nm, 488 nm, 561 nm, and 633 nm) FACSAria III flow cytometer, and collected on ice in sterile tubes. COP cells were defined as the CD45+/CD34+/ALP+ population during flow cytometry. The COP cells were then plated at a density of 1.25 × 10^5^ on fibronectin-coated tissue culture flasks in low-glucose Dulbecco’s modified eagle medium (DMEM), supplemented with 15% FBS, 1% penicillin/streptomycin and 2.5 mM l-glutamine. The cells underwent 2 passages until adequate cells had been collected for the transcriptomic and proteomic analyses.

### MPCs

A 5–8 ml of red bone marrow was collected via aspiration from the vertebral bodies of orthopedic surgery patients at The Medical University of South Carolina under the approval of the Institutional Review Board. The bone marrow was collected into EDTA-coated tubes and then passed through a 100 μm filter to remove bone debris and cell aggregates. The bone marrow then underwent density gradient separation before incubation with a magnet conjugated CD271 antibody for 30 minutes at room temperature. The cells were then passed through a magnetic column with the CD271 + MPCs isolated as characterized previously (15). The MPCs were then cultured in DMEM with the same supplements described earlier. For 2 passages, until adequate cell numbers had been acquired.

### ADSCs

Primary ADSCs from liposuction aspirated were acquired commercially for research use (LACell, New Orleans, LA, USA) as previously described (20). Only samples from nonobese, healthy donors were used in the analyses. The ADSCs were thawed into DMEM as described earlier and passaged twice to gain adequate cell numbers for the analysis.

### Transcriptomic Analyses

#### RNA isolation

Following 2 passages in cell culture, COP cells, MPCs, and ADSCs were washed twice with PBS and trypsinized before undergoing RNA extraction with the QIAGEN miRNEasy Minikit (QIAGEN, USA) according to manufacturer instructions. RNA purity and integrity were evaluated by Qubit fluorometer (Invitrogen, Waltham, MA, USA) and Agilent TapeStation electrophoresis, with all RNA used in the analysis having a RIN > 9. A secondary assessment of RNA integrity was performed with an AATI fragment analyzer to ensure no degradation in processing and transport.

#### RNA sequencing

Sequencing was performed at the Micromon Genomics Institute at Monash University, Melbourne, Australia. Libraries were prepared with the MGIEasy RNA chemistry system, and sequencing was performed on an MGITech MGISEQ2000RS system. Sequencing was conducted over 3 sequencing lanes, with greater than 400 million raw reads per lane. Reads were mapped to the human genome index file from the University of California, Santa Cruz, CA, USA (March 2021) with the “Rsubread” package on R (v4.0.5). Phred scores were calculated, with scores >30 deemed adequate for analysis.

#### Proteomics

Second passage cells were collected by trypsinization and washed 3 times with PBS to remove contaminant protein. Cells were then lysed by agitation in a 9 M urea, 50 mM Tris-HCl, with 100 units/ml of nuclease at pH8 for 30 minutes, then centrifuged at 20 000*g* for 15 minutes. Protein was then reduced in dithiothreitol (1 mM), alkylated in iodoacetamide (5 mM), and digested with Lys-C at a 1:50 ratio of protease to protein for 3 hours, then overnight in trypsin at 37 °C at the same ratio. The digestion was then acidified with 1% formic acid and desalted with C18 stage tips conditioned with 5% formic acid and 80% acetonitrile before being dried in a SpeedVac.

#### Mass spectrometry

The purified peptides were analyzed via label-free proteomics on an EASY nLC 1200 System (ThermoScientific, Waltham, MA) in line with the Orbitrap Fusion Lumos Tribrid Mass Spectrometer (ThermoScientific) (control software v. 4.2.28.14). A 2 µg of peptides were loaded on C18 reversed-phase column (Acclaim PepMap RSLC, 75 µm × 50 cm (C18, 2 µm, 100 Å) ThermoFisher cat. # 164536) using a 5% to 40% B gradient in 180 minutes (Solvent A: 5% acetonitrile/ 0.1% formic acid; Solvent B: 80% acetonitrile/ 0.1% formic acid) at a flow rate of 300 nL/minute.

Spectra were acquired with a high resolution (60 000) Fourier transform mass spectroscopy survey scan in data-dependent mode, with a mass range of 375–1 500 m/z, followed by 3s cycle time tandem mass spectra (MS/MS) of the most intense precursors. Higher energy collision dissociation (HCD) fragmentation was performed with a precursor isolation window of 1.6 m/z, a maximum injection time of 50 ms, and an HCD collision energy of 35%. Precursors within 10 ppm mass tolerance were dynamically excluded from resequencing for 15 seconds. Precursor ions with charge states that were undetermined, 1 or >5 were excluded.

#### Mass spec processing

The spectra acquired were searched through the MaxQuant platform (v.1.6.3.3) and normalized with the label-free quantification (LFQ) algorithm. Data was matched to the SwissProt database (March 2021), and a database of contaminants. False discovery rate (FDR) was calculated through a reverse database strategy, set at 1% at protein and peptide levels. Peptides were required to be fully tryptic, and of at least 7 residues with lysine-proline cleavage. A maximum of 2 missed cleavages were permitted. The MaxQuant results were then analyzed in Perseus (21). Proteins identified by a single modified peptide, contaminants, and reverse-matched peptides were removed, and LFQ protein intensities log_2_ transformed. Protein intensities were visualized, and normalized, and ultimately had similar distribution.

#### Statistical analysis

Analyses were performed at the Bioinformatics Platform of the Research Institute of the McGill University Health Centre (Montreal, Canada). Lowly expressed genes were filtered using edgeR, and library sizes and distribution were visualized and normalized. Normalization was also performed to limit composition bias. Finally, differential expression analyses for prespecified contrasts (younger donors vs older donors) with limma-voom using contrast analysis and moderated Bayesian statistics. Comparisons with a FDR < 0.05 were considered significant. Additional pathway and gene ontology analyses were performed to identify functional changes within the data sets using SRPlot (22). The raw data for this study is publicly available at the Sequence Read Archive with the accession number PRJNA987312 (sequencing data), and the mass spectrometry proteomics data have been deposited to the ProteomeXchange Consortium via the PRIDE partner repository with the data set identifier PXD035803.

## Results

### In Stem Cells Isolated From Older Individuals, Differentiation and Proliferation Genes Are Differentially Expressed but Not Proteins

**Figure 1.**
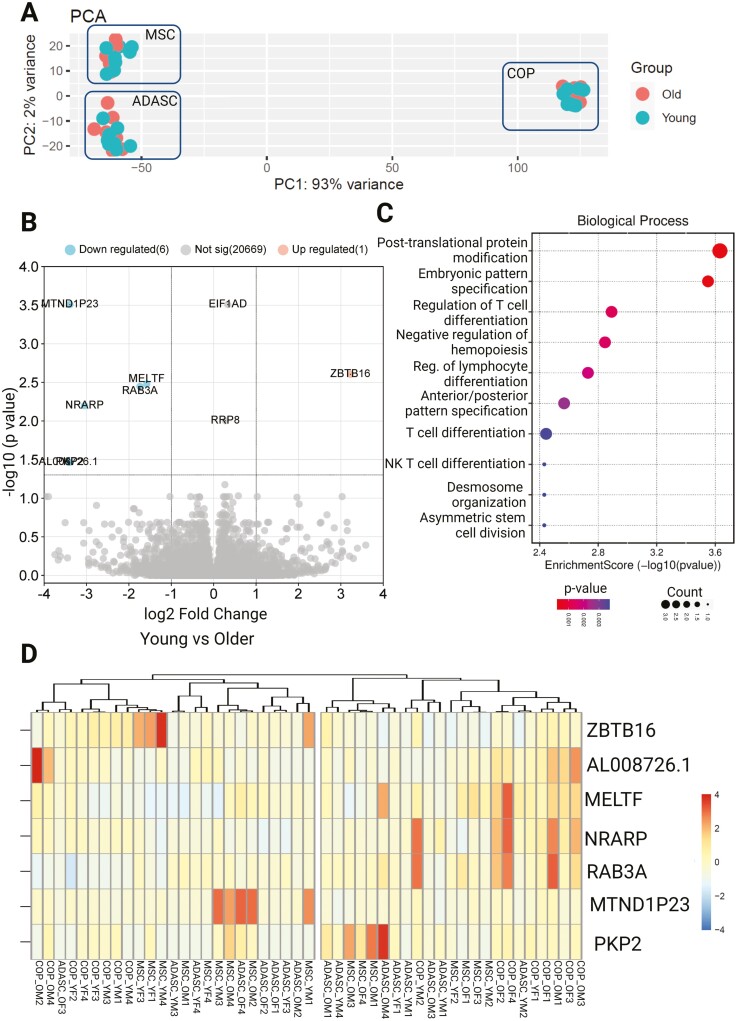
Differential expression of genes across cells grouped by age. (**A**) PCA plot showing clustering by cell type, rather than age, (**B**) Volcano plot showing differential expression of younger samples, compared to older, (**C**) Biological process enrichment analysis, (**D**) Heatmap showing differentially expressed genes. ADASC = Adipose-derived adult stem cells; COP = Circulating osteoprogenitor cells; OM = Older Male; OF: Older Female; MPC = Mesenchymal stem and progenitor cells; YM = Young Male; YF: Young Female.

**Figure 2.**
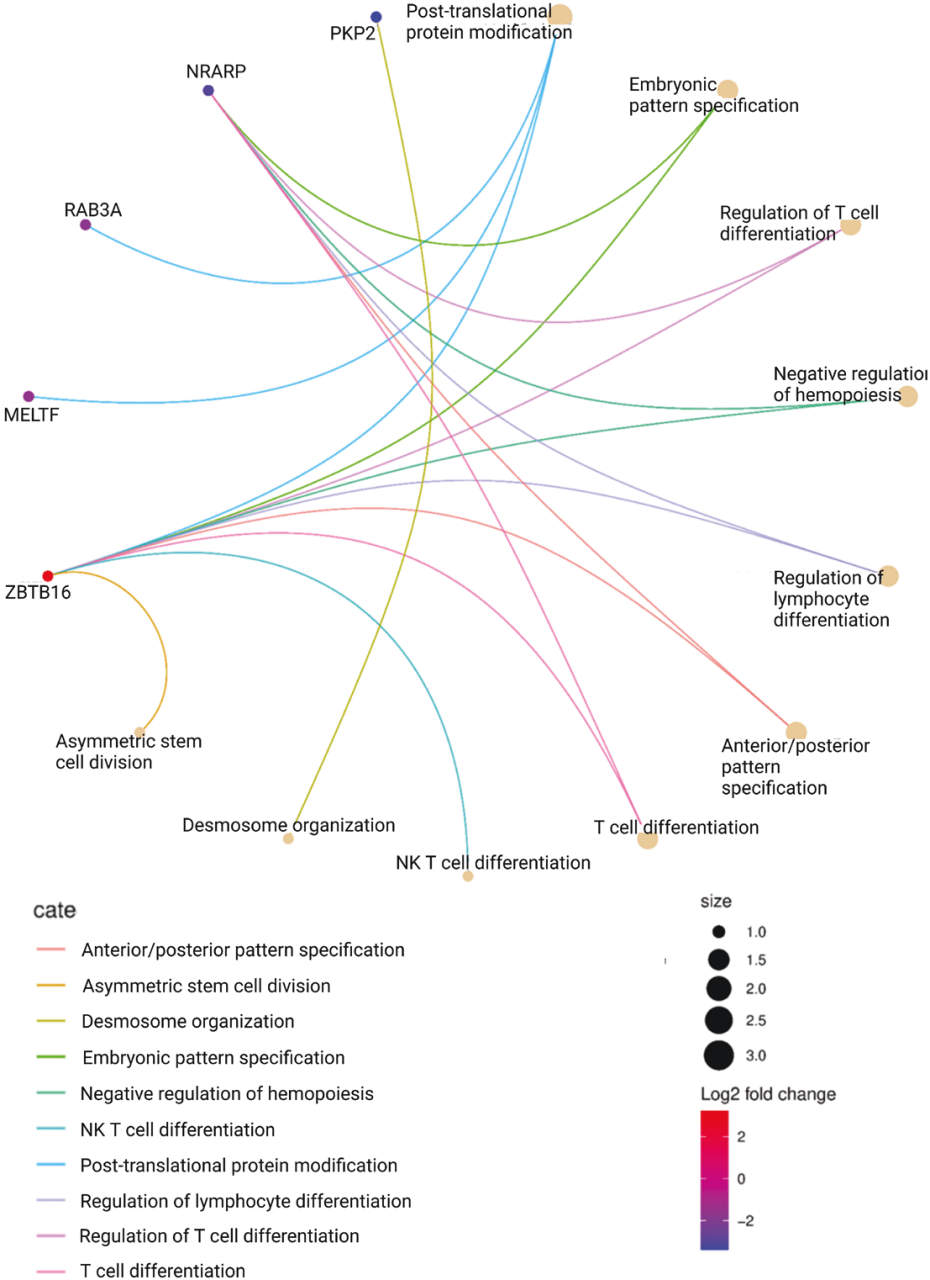
Circular network plot showing the differentially regulated genes and their biological process ontologies.

In principle component analyses based on the transcriptome, the samples clustered by cell type rather than age, with distinct localization of MPC, ADASC, and COP cells (Figure 1A). Differential expression analyses revealed 7 differentially expressed genes (DEGs) common across the 3 cell types (Figure 1B and D). Pathway and ontology analysis of the DEGs showed significant enrichment in pathways associated with differentiation, immune activation, stem cell division, and embryonic pattern specification (Figures 1C and 2, Supplementary Table 1). None of these translational changes were preserved at a proteome level, with no differentially expressed proteins across the age groups.

### Differential Expression of Genes and Proteins in MPCs From Older and Younger Donors

**Figure 3.**
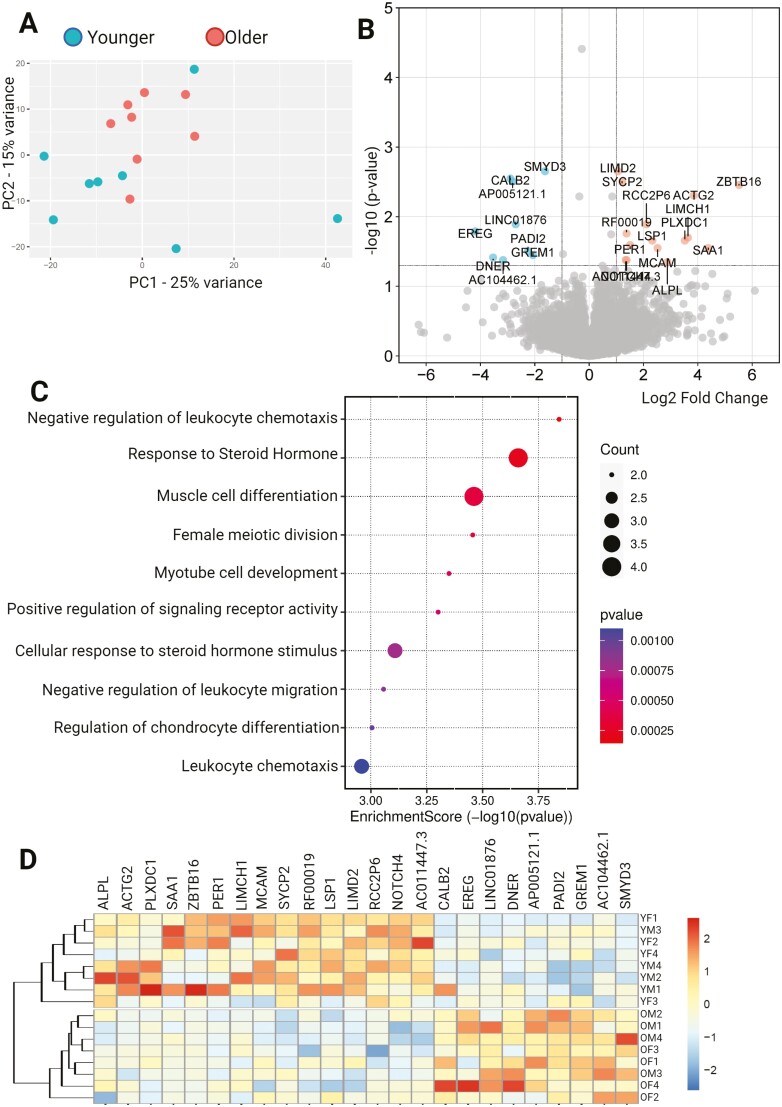
Transcriptome analysis of MPCs. (**A**) PCA plot showing limited clustering of samples by age, (**B**) Volcano plot showing differentially expressed genes in younger donors versus older, (**C**) Biological process ontology enrichment analysis of differentially expressed genes, and (**D**) Clustered heat map showing differentially expressed genes across the data set. ADASC = Adipose-derived adult stem cells; COP = Circulating osteoprogenitor cells; MPC = Mesenchymal stem and progenitor cells; OM = Older Male; OF = Older Female; YM = Young Male; YF = Young Female.

**Figure 4.**
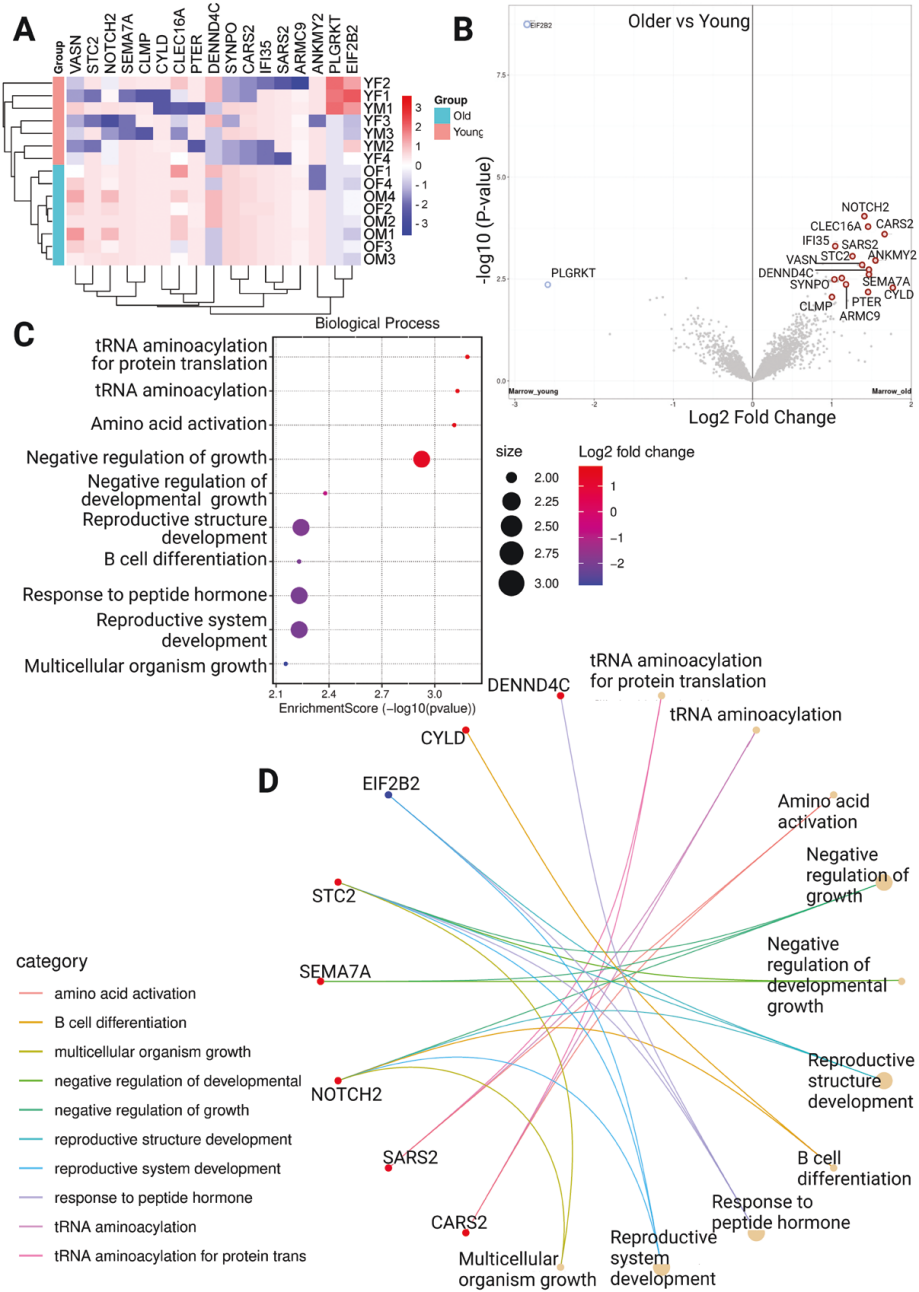
Proteomic analysis of MPCs (**A**) Heatmap showing differentially expressed proteins between older and younger donors, (**B**) Volcano plot showing differentially expressed proteins between younger and older, (**C**) Biological process ontology analysis showing enrichment of pathways in the set, (**D**) Circular network plot showing proteins and their associations with the enriched pathways. ADASC = Adipose-derived adult stem cells; COP = Circulating osteoprogenitor cells; MPC = Mesenchymal stem and progenitor cells; OM = Older Male; OF = Older Female; YM = Young Male; YF = Young Female.

Analysis of the bone marrow MPCs alone showed greater differential expression between older and younger samples, with both transcriptomic and proteomic changes. In principle component analyses, there was limited clustering of cells along any dimension (Figure 3A). There were 24 DEGs across the 2 age groups, with 9 genes under-expressed in younger samples and 15 genes over-expressed (Figure 3B and D). Biological process ontology analysis showed significant enrichment of pathways associated with muscle differentiation and development, cell division, response to steroid hormones, regulation of chondrocyte differentiation, and immune system activation and migration (Figure 3C). At a protein level, there were 17 differentially expressed proteins (Figure 4A), with 2 proteins under-expressed and 15 over-expressed in older donors versus younger donors (Figure 4B). Biological process ontology analysis revealed the enrichment of genes associated with the regulation of growth, tRNA aminoacylation, and hormone responses in the differentially expressed protein set (Figure 4C and D).

### Differentially Expressed Genes, but not Proteins in COP Cells and ADSCs

**Figure 5.**
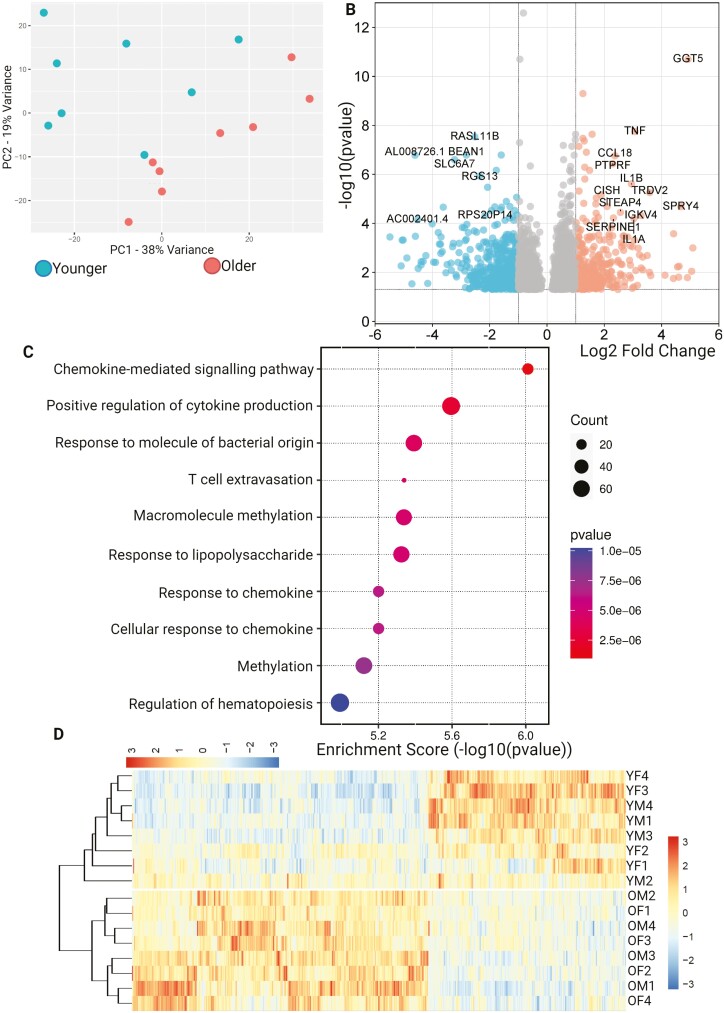
Differential expression of genes across COP cells grouped by age. (**A**) PCA plot showing limited clustering by age, (**B**) Volcano plot showing differential expression of younger samples, compared to older, (**C**) Biological process enrichment analysis, (**D**) Heatmap showing differentially expressed genes. ADASC = Adipose-derived adult stem cells; COP = Circulating osteoprogenitor cells; MPC = Mesenchymal stem and progenitor cells; OM = Older Male; OF = Older Female; YM = Young Male; YF = Young Female.

The transcriptome of COP cells showed some level of clustering on PCA along both PC1 and PC2. However, this was inconsistent, and the separation of age groups was poor (Figure 5A). The transcriptome of COP cells has a large number of differentially expressed genes between older and younger samples, with 473 under-expressed and 315 over-expressed genes in younger individuals versus older people (Figure 5B and D). Biological process ontology analysis showed enrichment in several pathways associated with immune system activation and reactivity, as well as macromolecule methylation (Figure 5C). Despite the large number of DEGs, there were no consistently regulated proteins in the older COP cell samples versus the younger ones.

**Figure 6.**
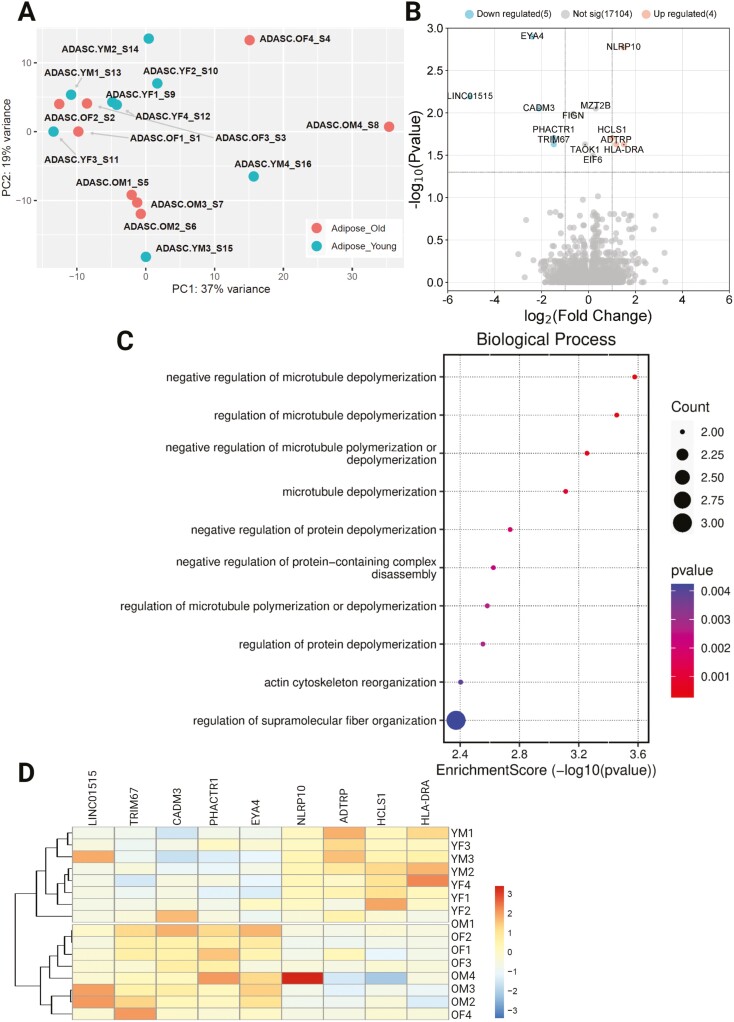
Differential expression of genes across ADSCs grouped by age. (**A**) PCA plot showing no clustering by age, (**B**) Volcano plot showing differential expression of younger samples, compared to older, (**C**) Biological process enrichment analysis, (**D**) Heatmap showing differentially expressed genes. ADASC = Adipose-derived adult stem cells; COP = Circulating osteoprogenitor cells; MPC = Mesenchymal stem and progenitor cells; OM = Older Male; OF = Older Female; YM = Young Male; YF = Young Female.

The transcriptome of ADSCs also showed no apparent clustering on PCA by age (Figure 6A), and there were far fewer differentially expressed transcripts, with 5 under-expressed and 4 over-expressed in younger donors versus older donors (Figure 6A and D). Biological process ontology analysis of the DEGs showed that pathways related to cytoskeletal rearrangement were largely enriched across the sample. As with COP cells, no differentially expressed proteins existed between the younger and older samples.

## Discussion

For mesenchymal progenitor populations to realize their potential for clinical utilization, a solid understanding of how their physiology changes over the life span is critical. In this work, we compared the transcriptome and proteome of primary MPCs, COP, and ADSCs from older adults to that of younger adults to identify key mechanisms of stem cell aging. Across all samples, there were a greater number of changes seen in the transcriptome, but broadly, these did not carry through into the proteome of the cells, except for in the MPCs. Only older MPCs had a distinctive proteomic phenotype with several differentially regulated proteins, which may have clinical implications.

The primary goal of this work was to identify whether there were overarching transcriptomic or proteomic changes that typified an older stem and progenitor cell, which may serve to understand the mechanisms of age-related stem cell exhaustion and identify therapeutic or prognostic targets in the management of key degenerative diseases associated with aging. Interestingly, the transcriptome of the pooled stem cells did show a set of consistent changes across the older progenitor cells, irrespective of cell type. The only gene under-expressed in older adults compared to younger ones was the zinc finger and BTB domain containing 16 (ZBTB16), a nuclear transcription factor involved in regulating cell cycle progression, which is a key regulator of stem cell self-renewal and differentiation (23). A decrease in this gene may underpin several alterations in differentiation seen in stem cells from older adults. It has been shown that ZBTB16 is highly expressed in undifferentiated stem cells with a high capacity for proliferation and then downregulated as a stem cell becomes terminally differentiated (23). In addition, ZBTB16 has been shown to be a key regulator of osteoblastic differentiation in MPCs, again suggesting its suppression could lead to altered regeneration of bone (24).

Additionally, there was a significant decrease in melanotransferrin (MELTF) expression in older stem cells across all 3 groups. MELTF (also known as CD228) is a membrane-bound transferrin, first associated with melanoma development. More recently, it has been found to be expressed in bone marrow MPCs, and interestingly, its inhibition was associated with both increased adipo- and osteogenesis (25). There was also an increase in the expression of the Ras-related protein RAB3A, plakophilin 2 (PKP2), and NOTCH-regulated Ankyrin Repeat Protein (NRARP) genes, as well as the MTND1P23 pseudogene. The RAB3A and NRARP genes have never been associated with mesenchymal progenitor function. RAB3A is a key mediator of neuronal exocytosis of neurotransmitters that has been well studied in neurological tissues, although it is unclear what its role in mesenchymal progenitor may be (26). There is, however, significant cross-over seen between neuronal and mesenchymal progenitors from a number of sources (27–30). The NOTCH signaling pathways have a substantial role in stem cell differentiation, associated with muscle homeostasis (31), bone formation (32), and adipogenesis (33). In muscle tissue, while NRARP is upregulated in response to NOTCH signaling, it is a negative regulator—reducing the ultimate formation of tissue (34).

PKP2 is associated with cardiac muscle cell differentiation in normal contexts, mutations in the gene causing decreased contractility and desmosomal structure in arrhythmogenic cardiomyopathy (35), however, it has never been evaluated in mesenchymal progenitors. Although many of these transcriptomic changes suggest potential effects on physiologies tied to stem cells, it is notable that none of these were carried through to the proteome. This may be due to the critical role of local environmental factors in the stem cell niche, which drives a significant portion of stem cell behavior (36). Throughout fetal, infant, and adolescent development, the stem cell niche and the progenitors coregulate, leading to the development of distinct populations of adult stem cells (36). It is possible that while there may be underlying genetic changes contributing to the decline in stem cell function, the disparity in the environment of bone marrow MPCs, COP cells, and ADSCs leads to different phenotypes of expressed protein.

When analyzed separately, only the bone marrow MPCs showed significant changes in both the transcriptome and proteome. The most notable changes seen in the transcriptome of the MPCs were in genes associated with steroid hormone response and muscle differentiation. The genes related to steroid hormone response over-expressed in older stem cells were Peptidyl Arginine Deiminase, Type II (PADI2), and SET and MYN-domain containing 3 (SMYD3). PADI2 has a role in the maintenance of stem cell proliferation and differentiation, with increased expression leading to greater proliferation, possibly suggesting promotion of stem cell function (37). However, overexpression of PADI2 in bone marrow MPCs leads to increased interleukin-6 (IL6) expression, a key inflammatory mediator (35). In recent years, the immune role of bone marrow MPCs has gained prominence, and it is now known that they contribute to and, in turn, are influenced by the chronic inflammation associated with aging (38). This overexpression of PADI2 could represent either a mechanism driving proinflammatory change or a response to chronic inflammation in the bone marrow. SMYD3 is a chromatin-modifying oncogene overexpressed in cancer stem cells, where it promotes proliferation, regulates the cell cycle, and mediates immortalization of cancer cells (39). In development, over-expression of SMYD3 regulates mesodermal tissue fate of embryonic stem cells (40), however, little is known about its role in adult mesenchymal progenitors.

In addition to the over-expression of SMYD3 and PADI2, both the period circadian regulator 1 (PER1) and alkaline phosphatase biomineralization associated (ALPL) genes were under-expressed in older cells. ALPL, the subvariant of alkaline phosphatase related to the bone, liver, and kidney, is strongly associated with osteogenesis and mineralization in MPCs, and its under-expression is highly suggestive of reduced capacity for osteoblastic differentiation (41,42). In addition, ablation of the ALPL gene in MPCs induces characteristics of bone aging, including impaired osteogenesis and lipid accumulation, further strengthening its mechanistic role (41).

The role of PER1 in aging MPCs is less clear. PER1 is one of the essential mechanistic drivers of the circadian rhythm, being cyclically expressed on an approximately 24-hour cycle in the suprachiasmatic nucleus, where it is a master regulator of chronobiology in a range of tissues throughout the body, affecting the sleep-wake cycle, appetite, and energy metabolism, and cell cycle control (43). However, PER1 has also been shown to have diverse roles in the physiology of several stem cells, including embryonic, ADSC, MPCs, and dental pulp stem cells (44). In mesenchymal progenitor populations, it has been shown to have significant effects on the differentiation of the cells, with osteoblastic, chondrogenic, and adipogenic capacity affected by expression of PER1, or its downstream factors such as BMAL1 (45), with PER1/BMAL1 knockout animals having increased bone volume (46).

Although there is a well-known decrease in muscle anabolism in older adults, counterintuitively, the 4 differentially expressed genes associated with muscle cell differentiation Gremlin1 (GREM1), SMYD3, Delta/Notch-like epidermal growth factor (EGF)-related receptor (DNER), and epiregulin (EREG), were all overexpressed in older MPCs. The direct role of bone marrow MPCs in muscle repair and regeneration is controversial. Although bone marrow MPCs can differentiate to form skeletal muscle, this is unlikely to occur directly in vivo, with muscle having a dedicated reserve of lineage-restricted satellite cells that serve to regenerate and repair tissue (47). However, while these genes are associated with muscle development in satellite cells, GREM1 expression also identifies stem cells in bone, promoting osteoblastogenesis via RUNX2 expression (48), and DNER promotes proliferation through PI3K/AKT signaling in cancer stem cells (49), suggesting other potential roles for the genes outside skeletal muscle.

At the proteomic level, the most prominent ontology represented in the differentially expressed proteins was negative regulation of growth, with the staniocalcin2 (STC2), Semaphorin7a (SEMA7A), and NOTCH2 proteins all overexpressed in older MPCs. STC2 is an antiapoptotic, antioxidative protein secreted by MPCs in response to inflammation, where it inhibits the NLRP3 inflammasome (50,51). STC2 enhances stem cell survival by reducing oxidative stress, and its expression in the older primary cells is likely a response to inflammation rather than the cells themselves being inherently anti-inflammatory compared to younger cells (51,52). Overexpression of SEMA7A in MPCs has also been linked to the inflammatory and oxidative stress response, promoting the secretion of the key anti-inflammatory interleukin 10 from resident macrophages (53). NOTCH2 is a well-known factor involved in both immune and musculoskeletal tissue development. Increased NOTCH2 expression has been shown in MPC isolated from geriatric mice, in whom there was greater adipogenic and poorer osteogenic differentiation (54). It has also been demonstrated that MPCs express NOTCH2 to induce the accumulation of regulatory dendritic cells (DCs) in order to curb inflammation in response to lipopolysaccharides (55). Taken together, these genes appear to indicate that MPCs from older individuals are under greater oxidative and inflammatory stress, which is likely to affect terminal differentiation and tissue formation.

The changes to expression in COP cells were less clear than in the bone marrow MPCs, with a large number of differentially expressed genes in the transcriptome but no consistent proteomic phenotype. The clear change to the transcriptome of COP cells in older adults is a shift to proinflammatory signaling, with overexpression of key inflammatory mediators such as interleukin 1 (IL1)-beta, IL1-alpha, and tumor necrosis factor, as well as important chemotactic factors CCL18, and GGT5. COP cells have been shown to have a more prominent immune role than bone marrow MPCs, and ADSCs (56), and thus it stands to reason that they may be more strongly affected by inflammaging and chronic oxidative stress with advancing age. In addition, given their native environment in the circulation, they are likely exposed to a wide range of factors reflective of the general condition of the individual. Given the well-known increase in chronic inflammation in older age, it is likely that COP cells are regulated into a proinflammatory state and then, in turn, drive further chronic inflammation. Whether this leads to direct impacts on musculoskeletal tissues as COP cells are home to locations such as bone is unknown. Like COP cells, the bone-resorbing osteoclast is a monocyte lineage cell sensitive to inflammation, which drives osteoclastogenesis and catabolism of mineralized bone. It has been suggested that COP cells may mediate a link between the bone and immune systems, regulating both osteoclast and osteoblast within the bone microenvironment, and the proinflammatory shift seen in the older cells in this study may drive the acceleration of bone loss in aging. The diversity in the microenvironment of COP cells in different populations may also explain the lack of a consistent proteomic phenotype. Significant external stimulus input leads to a large amount of post-translational modification and regulation, likely leading to considerable variance in the ultimately expressed proteome of the cells.

A similar expression pattern was seen in the ADSCs, with a small number of differentially expressed genes but no consistent proteomic phenotype in the older cells. Most changes in the transcriptome were centered on cytoskeleton regulation, immune response initiation, cell movement, and adhesion. Older ADSCs had increased expression of the LINC101515 noncoding RNA, as well as CADM3, PHACTR1, TRIM67, and EYA4. These genes have not been evaluated in mesenchymal progenitors, and so the significance of these changes is unclear. However, increased expression of PHACTR1 has been shown to increase mineralization within endothelial progenitors and is known to be involved in cell mobility, apoptosis, and matrix remodeling (57,58), though whether this is the case in ADSCs requires further research. As with COP cells, there was also no evident phenotype in the proteome of the ADSCs. This may again be due to the variety of changes accruing in the vascular microenvironment in the adipose tissue of older adults, based on health status. Adipose tissue strongly regulates a range of chronic diseases, and the profile of the local stem cells is likely to reflect this.

The major strength of this study is its scale. There is no comparable work using primary harvested stem cells across as many individuals, providing significant power to detect changes across the data set. In addition, robust data management, cell treatment and analysis, with all samples grown and analyzed simultaneously, provide confidence in the findings. The major challenge with in vivo generalizability in these results is the expansion of the cells in culture. In an ideal setting, cells would be harvested and analyzed directly; however, due to the scarcity of the cells, expansion is required to get significant volumes of protein and RNA. Expansion was minimized to ensure the smallest amount of variability between the data gained here and what is likely the case in vivo. In addition, the lack of broader health information from the donors makes some of the changes, or lack thereof, challenging to contextualize. All donors were cleared of major diseases before cell isolation, however, the addition of general health indicators, biomarkers of inflammation, metabolic disorders, or other key physiological indicators associated with the disease would improve generalizability.

In conclusion, in this study, we used a geroscience approach to identify a range of genetic and proteomic changes occurring with aging in mesenchymal stem cells. This understanding will have important implications for ongoing work in stem cell therapeutics for musculoskeletal diseases in older adults, allowing for improved screening, stimulation, and individualization of treatments. Of note is the proinflammatory shift of stem cells taken from older adults and a pattern of altered differentiation status in all 3 stem cell types. This has significant implications for treating specific diseases; for example, COP cells harvested from older adults may not be suitable for inflammatory diseases such as osteoarthritis, and autologous MPCs may not be ideal for muscular applications. These results should allow for more effective individualized treatments for age-related conditions, improving outcomes. Future research should build on this work by investigating the influence of chronic disease on the physiology of stem cells in older adults, as well as exploring the roles of the identified differentially expressed genes and proteins from this study, many of which have not been adequately explored in mesenchymal progenitor populations.

## Supplementary Material

glae147_suppl_Supplementary_Material

glae147_suppl_Supplementary_Data
