# Supplementary material for: Differential Responses to Aging Among the Transcriptome and Proteome of Mesenchymal Progenitor Populations
Source: J Gerontol A Biol Sci Med Sci. 2024 Jun 5;79(9):glae147. doi: 10.1093/gerona/glae147 (PMC11369222; doi:10.1093/gerona/glae147)
Supplement: glae147_suppl_Supplementary_Material [file glae147_suppl_supplementary_material.docx]

| **Cell type** | **Age**  ***Mean (SD)*** | |
| --- | --- | --- |
|  | **Young** | **Older** |
| **MPC** | 28.13(4.73) | 59.63 (5.57) |
| **ADSC** | 27.25 (6.04) | 59.38 (6.30) |
| **COP** | 27.63 (5.70) | 59.88 (3.79) |

**Supplemental Table 1**
